# Long Noncoding RNAs in Neurodegenerative Diseases: Pathogenesis and Potential Implications as Clinical Biomarkers

**DOI:** 10.3389/fnmol.2021.685143

**Published:** 2021-08-04

**Authors:** Meng Zhang, Ping He, Zhigang Bian

**Affiliations:** ^1^Department of Gerontology and Geriatrics, Shengjing Hospital of China Medical University, Shenyang, China; ^2^Department of Otolaryngology Head and Neck Surgery, Shengjing Hospital of China Medical University, Shenyang, China

**Keywords:** biomarkers, circulating, lncRNA, miRNA, neurodegenerative disease

## Abstract

Neurodegenerative diseases (NDDs), including Alzheimer’s disease (AD), Parkinson’s disease (PD), Huntington’s disease (HD), and amyotrophic lateral sclerosis (ALS), are progressive and ultimately fatal. NDD onset is influenced by several factors including heredity and environmental cues. Long noncoding RNAs (lncRNAs) are a class of noncoding RNA molecules with: (i) lengths greater than 200 nucleotides, (ii) diverse biological functions, and (iii) highly conserved structures. They directly interact with molecules such as proteins and microRNAs and subsequently regulate the expression of their targets at the genetic, transcriptional, and post-transcriptional levels. Emerging studies indicate the important roles of lncRNAs in the progression of neurological diseases including NDDs. Additionally, improvements in detection technologies have enabled quantitative lncRNA detection and application to circulating fluids in clinical settings. Here, we review current research on lncRNAs in animal models and patients with NDDs. We also discuss the potential applicability of circulating lncRNAs as biomarkers in NDD diagnostics and prognostics. In the future, a better understanding of the roles of lncRNAs in NDDs will be essential to exploit these new therapeutic targets and improve noninvasive diagnostic methods for diseases.

## Introduction

Neurodegenerative diseases (NDDs) are characterized by the loss of neuronal cells in the nervous system (Rittiner et al., 2020). Common NDDs include Alzheimer’s disease (AD), Parkinson’s disease (PD), Huntington’s disease (HD), and amyotrophic lateral sclerosis (ALS; Ang et al., 2010). Currently, no effective treatments are available for NDDs, thus posing a heavy economic burden on society, families, and individuals, and severely affecting the lifestyle and health of patients and caregivers (Liao et al., 2019). Studies have identified a variety of pathophysiological mechanisms involved in NDD pathogenesis, including oxidative stress, mitochondrial dysfunction (Lin and Beal, 2006), excitotoxic amino acids (Garcez et al., 2019), and inflammatory reactions (Chen et al., 2016). Despite decades of research, the causes of NDDs remain unknown. In recent years, noncoding RNA (ncRNA) has gained importance as a major research direction in the study of NDD pathogenesis (Riva et al., 2016). In patients with AD compared with healthy controls, the antisense transcription of BACE1 is up-regulated, thus leading to increased BACE1 protein expression. Similarly, overexpressed BACE1 increases levels of Aβ peptide, the main component of amyloid plaques (Koelsch, 2017). In other research, RNA sequencing from white blood cells has shown lower expression of 13 lncRNAs in patients with PD than in healthy controls, thereby providing an experimental basis for further lncRNA research on PD pathogenesis (Soreq et al., 2014). Although the roles of ncRNAs in NDDs are difficult to fully elucidate, studies are increasingly showing that ncRNAs play important roles in both normal and pathological neurobiology (Maoz et al., 2017; Wu and Kuo, 2020).

Currently, commercially viable biomarkers for wide use in the early diagnosis of NDDs in clinical practice are lacking. In addition, although many peripheral blood biomarkers for predicting disease prognosis, treatment, and outcome have been evaluated at different disease stages, they have not yet entered clinical trials for evaluation. In recent years, circulating Aβ_42_ and Aβ_40_, α-synuclein (α-syn), transactive response DNA-binding protein of 43 (TDP-43), and other body fluid biomarkers from the blood or urine have emerged (Atik et al., 2016; Feneberg et al., 2018; Palmqvist et al., 2019); however, they remain far from clinical application.

lncRNAs generally refer to ncRNA transcripts >200 nucleotides (nt) in length. Researchers identified these noncoding sequences as early as the 1970 s. According to the prevailing understanding of evolutionary biology at the time, lncRNAs were predicted to have no biological function and were therefore deemed “junk DNA.” However, as research advanced, studies increasingly suggested that this “junk DNA” has important biological functions (Chen Y. et al., 2019). In fact, lncRNAs exert their biological functions by acting as signaling molecules, protein complex scaffolds, and/or molecular decoys to enhance gene transcription (Yang et al., 2019). Similarly, lncRNAs play important roles in the development and differentiation of the central nervous system (CNS). Deep sequencing data from tissues and cells have indicated that lncRNAs have stronger cell and tissue specificity than coding RNAs (Kozomara and Griffiths-Jones, 2014). A recent brain development study using RNA-seq, chromatin immunoprecipitation-sequencing, and lncRNA and mRNA cluster analysis, has observed that lncRNA is more tissue-specific than mRNA (Ramos et al., 2013). In addition, the number of lncRNAs far exceeds that of coding genes, thus potentially enabling them to serve as disease diagnostic and prognostic biomarkers.

**Figure 1:**
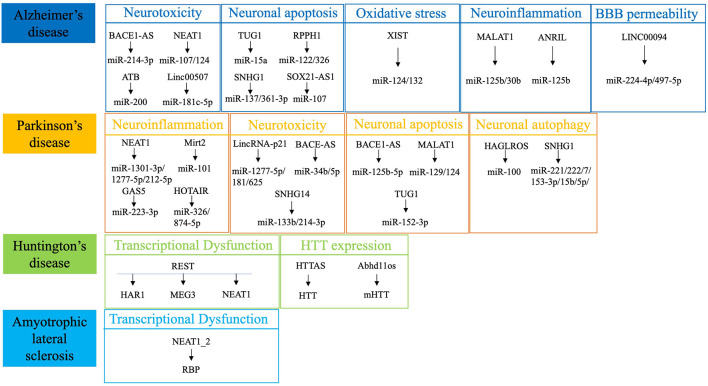
Summary of the most dysregulated lncRNAs in neurodegenerative diseases. REST, RE1-silencing transcription factor; HTT, Huntingtin; RBP, RNA-binding protein.

Studies investigating the involvement of ncRNAs in NDD pathogenesis have primarily focused on microRNAs (miRNAs), whereas the exact lncRNA mechanisms involved in NDD pathogenesis remain unclear (Riva et al., 2016; Haque et al., 2020). In addition, many studies have focused on circulating miRNAs as NDD biomarkers (Roser et al., 2018), but lncRNAs have not been fully evaluated. Compared with lncRNAs, miRNAs have been more widely studied as diagnostic biomarkers for NDDs (Kou et al., 2020; Zhao Y. et al., 2020), and several meta-analyses have suggested that miRNAs have clinical practical value as biomarkers for the diagnosis of neoplastic diseases (Zhou et al., 2018; Jayaraj et al., 2019). The stable presence of circulating miRNA in the serum, particularly after the elucidation of exosomes, has enriched the understanding and detection of circulating miRNA (Cha et al., 2019; Chen J. J. et al., 2019). Many databases for miRNA research are available, and studies on the mechanisms of miRNA involvement in NDDs are updated in a timely manner. In contrast, few clinical studies, particularly well-designed controlled clinical studies, have been conducted on lncRNAs; therefore, such research remains in the preliminary stages. Additionally, the slow updating of public databases that can be used to retrieve lncRNAs has restricted research progress on lncRNAs in NDDs (Maracaja-Coutinho et al., 2019). However, lncRNA research has rapidly emerged and provided growing evidence of lncRNAs as potential circulating diagnostic biomarkers in recent years. Herein, we review the involvement of lncRNAs in NDD pathogenesis (Figure 1), analyze their applications as biomarkers in clinical practice and discuss the inclusion of lncRNAs as novel therapeutic strategies for various NDDs.

## Overview and Classification of lncRNAs

lncRNAs are widely distributed in the cytoplasm and nucleus and are mainly derived from protein coding gene interruption, chromatin recombination, noncoding gene replication reverse transposition products, adjacent fragment duplication within noncoding RNA, and insertion of transposable factors into genes (Yang et al., 2017). Most lncRNAs are transcribed by RNA polymerase II and processed by splicing, 5′ capping, and 3′ polyadenylation (Xing and Chen, 2018). Studies have shown that lncRNA expression exhibited tissue, temporal and spatial specificity; i.e., lncRNAs were differentially expressed among tissues, and their expression levels varied among developmental stages in the same tissue or organ (Liu et al., 2018). With the increase in interest in lncRNA research, several functions have been attributed to these molecules, including roles in gene transcription, splicing, protein translation, protein localization, stem cell pluripotency, and cell structural integrity (Engreitz et al., 2016). Depending on the relationship between lncRNAs and protein coding genes, these lncRNAs can be divided into intergenic, antisense, intronic, sense, and bidirectional categories. Similarly, depending on their implicated roles in molecular mechanisms, these lncRNAs can be divided into signal, decoy, guide, and scaffold molecules (Lee, 2012).

How lncRNAs are produced is unclear, but they have been suggested to have arisen through the following mechanisms: (i) in early evolutionary processes, the open reading frame of a protein-coding gene becomes mutated and gene structure is destroyed; (ii) after chromatin rearrangement, two un-transcribed and distant sequences merge and produce a multi-exon lncRNA; (iii) noncoding genes are produced by reverse translocation; (iv) two consecutive repetitive events generate lncRNAs with repetitive sequences inside ncRNAs; and (v) transposable components insert into genes and produce lncRNAs. Most lncRNAs exhibit specific tissue and cell expression during tissue differentiation and development and similarly have characteristic expression patterns in CNS diseases (Ransohoff et al., 2018; Sarropoulos et al., 2019).

## lncRNA Regulatory Mechanisms

lncRNAs are typically involved in cell function regulation as secondary or tertiary structures in functions such as DNA replication, RNA transcription, protein translation, cell proliferation, and differentiation (Robinson et al., 2020). The structural characteristics of lncRNAs facilitate their complex and precise regulatory roles in gene expression, with functions such as epigenetic phenomena including gene silencing, DNA methylation, histone modification, dormant transposon activation, RNA editing, and gene methylation (Johnsson et al., 2014). As new epigenetic regulatory molecules, lncRNAs have been implicated in epigenetic regulation (Heo et al., 2013). Gene transcription is a highly complex biological process, and lncRNAs appear to regulate gene transcription by mimicking DNA elements, competitively binding transcription factors, or conducting variable splicing (Wang et al., 2008). During gene expression, post-transcriptional gene regulation requires a series of post-transcriptional processes including gene processing, modification, and regulation, through incorporating RNA splicing, processing, splicing, maturation, metabolism, and stability regulation (Filipowicz et al., 2005). In an important step in mRNA processing and metabolism, the shearing of mRNA precursors is often regulated by lncRNAs.

Several studies have proposed that lncRNAs have pathophysiological roles in regulating miRNA expression (Huang, 2018). Because of the unique length characteristics of lncRNA molecules, miRNA recognition action sites have been identified along lncRNAs. These sites specifically bind miRNAs, thereby affecting *in vivo* lncRNA stability, mediating lncRNA degradation, and regulating cell biological functions (Huang, 2018). In addition, beyond acting as protein bait molecules that regulate target gene expression, lncRNAs also act as bait molecules for miRNAs: they adsorb miRNAs via target mimicry and inhibit further effects on target molecules. Moreover, lncRNAs may be used as molecular bait to indirectly regulate target gene expression by suppressing miRNA effects on their targets. lncRNAs also competitively bind mRNA to miRNA, thus directly regulating target gene expression (Wang et al., 2020a).

## NDD-Related lncRNAs

### AD-Related lncRNAs

AD is an NDD characterized by progressive cognitive decline and memory loss. Characteristic pathological manifestations include senile plaques, intracellular neurofibrillary tangles, and extracellular Aβ deposition. The disease etiology and pathogenesis have yet to be elucidated (Fernandez et al., 2019). In recent years, several genomic studies have shown that lncRNA expression was closely associated with AD pathogenesis (Idda et al., 2018).

Aβ is a polypeptide containing 39–43 amino acids, which is produced by proteolysis of the amyloid precursor protein (APP) via β- and γ-secretases (Zhou R. et al., 2020). Aβ deposition not only is associated with neuronal degeneration but also activates a series of pathological events. The polypeptide is the main cause of neuronal degeneration and cell death in AD brains (Sevigny et al., 2016). Faghihi et al. have shown that the antisense transcript of β-secretase (BACE1), i.e., lncRNA BACE1-AS, increased Aβ_1–42_ expression in patients with AD through a feed-forward regulatory mechanism (Table 1). BACE1-AS levels are elevated in patients with AD and in APP transgenic mice, thus suggesting that BACE1-AS levels may serve as a potential diagnostic biomarker and therapeutic target for AD (Faghihi et al., 2008). Liu et al. have constructed an AD senile plaque model by examining neuroblastoma cells (SH-SY5Y) with synthesized Aβ_1–42_, and down-regulating lncRNA BACE1-AS through siRNA silencing. The ability of BACE1 to shear APP and produce Aβ_1–42_ decreased, thus suggesting that lncRNA BACE1-AS is an important factor in Aβ_1–42_ formation (Liu et al., 2014). In addition, *in vivo* BACE1-AS knockdown mediated by lentiviral expression has been found to improve the memory and learning ability of SAMP8 mice, inhibit BACE1 and APP production, and prevent phosphorylation of the tau protein in mouse hippocampi (Zhang W. et al., 2018). He et al. have shown that BACE1-AS was up-regulated and miR-214-3p was down-regulated in the plasma from patients with AD as well as in SK-N-SH cells treated with Aβ. Additionally, BACE1-AS acts as a sponge for miR-214-3p, and miR-214-3p silencing reverses the effects of BACE1-AS knockdown on isoflurane-mediated apoptosis in Aβ-induced SK-N-SH cells. These data suggest that BACE1-AS aggravates isoflurane-induced neurotoxicity in AD via miR-214-3p sponging (He et al., 2020).

**Table 1:** Long noncoding RNAs associated with the pathogenesis of neurodegenerative diseases.

NDDs	lncRNA	Changes	Targets	Function	Reference
AD	BACE1-AS	Up in AD patients and in APP transgenic mice	Aβ; miR-214-3p	Increase_1–42_ expression through feedforward regulation; Aggravate neurotoxicity *via* sponging miR-214-3p	Faghihi et al. (2008) and He et al. (2020)
	NEAT1	Up in AD patients; Aβ-induced SH–SY5Y cells; AD mouse model	Aβ; miR-107; CDK5R1; miR-124; NEDD4L	Attenuate Aβ-induced neural injury; As a decoy of miR-107; Increased CDK5R1 levels by sponging miR-15/107 family; Increase miR-124 expression to perform neuroprotective effects; Promote PINK1 ubiquitination	Spreafico et al. (2018), Ke et al. (2019), Zhao et al. (2019); and Huang et al. (2020)
	MEG3	Down in AD rat model	Aβ	Inactivate the PI3/Akt pathway	Yi et al. (2019)
	BC1	Up in AD mice	FMRP	Induces APP mRNA translation via FMRP	Zhang T. et al. (2018)
	17A	Up in AD patients and AD cell models	Aβ	Enhance the secretion of Aβ *via* inducing inflammatory stimuli	Massone et al. (2011)
	MALAT1	Up in in PC 12 AD models	miR-125b; mR-30b	Sponge miR-125b to perform neuroprotective role; Serve as a sponge for mR-30b to up-regulate CNR1	Ma et al. (2019) and Li L. et al. (2020)
	ANRIL	Up in AD PC12 cells	miR-125a	Inhibit inflammatory cytokine expression	Zhou B. et al. (2020)
	XIST	Up in AD rat and cell model	miR-124; miR-132	Regulate BACE1 expression; Sponge miR-132 to perform neuroprotective role	Yue et al. (2020) and Wang X. et al. (2018)
	RPPH1	Up in AD mice	CDC42; miR-122; miR-326	Up-regulate the expression of CDC42; Activate Wnt/β-catenin signaling; Attenuate ERS and apoptosis induced by Aβ_25–35_	Cai et al. (2017) and Gu et al. (2020a,2020b)
	ATB	Up in AD patients and cell models	miR-200	Sponge miR-200 to perform neuroprotective role	Wang J. et al. (2018)
	EBF3-AS	Up in APP/PS1 mice	Aβ	Inhibit Aβ_25–35_ induced apoptosis	Gu et al. (2018)
	BC200	Up in AD cell model	BACE1	Suppress BACE1 expression	Li et al. (2018)
	SNHG1	Up in AD patients and SH–SY5Y cell models	miR-137; miR-361-3p	Exert neuronal protective effects by sponging miR-137; Promote cell injury	Wang et al. (2019) and Gao et al. (2020)
	TUG1	Up in AD mice	miR-15a	Suppress ROCK1 expression and inhibit neuronal apoptosis	Li X. et al. (2020)
	Linc00507	Up in APP/PS mice and SH–SY5Y cells	miR-181c-5p	Mediate tau protein hyperphosphorylation	Yan et al. (2020)
	SOX21–AS1	Up in SH–SY5Y cells	miR-107	Promote Aβ_1–42_ mediated *p*-Tau and neuronal apoptosis	Xu et al. (2020a)
	BDNF–AS	Up in PC 12 cells	BDNF	Regulate BDNF expression	Guo et al. (2018)
	LINC00094	Up in AD mice	miR-224-4p; miR-497-5p	Regulate BBB permeability in AD	Zhu et al. (2019)
PD	SNHG1	Up in PD patients and MPP+-induced cell	miR-221/222; miR-153-3p; miR-15b-5p; miR-7	Increase LC3-II levels and promote cytotoxicity; Aggravate MPP+-induced cellular toxicity; Contribute to Lewy body formation; Activate of NLRP3 inflammasome	Chen et al. (2016), Cao et al. (2018), Qian et al. (2019); and Zhao et al. (2020a)
	NEAT1	Up in PD mouse	PINK1; miR-1301-3p; miR-1277-5p; miR-212-5p	Inhibit the degradation of PINK1 protein; Regulate α-syn-induced activation of NLRP3 inflammation; Contribute to MPP^+^-induced neuron injury, inflammation and apoptosis	Yan et al. (2018), Sun et al. (2020); and Liu et al. (2020)
	HAGLROS	Up in PD mouse and MPP^+^-induced cell	miR-100	Regulate autophagy and apoptosis	Peng et al. (2019)
	BDNF-AS	Up in PD mouse and MPP^+^-induced cell	miR-125b–5p	Regulate autophagy and apoptosis	Fan et al. (2020)
	SNHG14	Up in PD mouse	miR-133b; miR-214-3p	Down-regulating α-syn; Regulate expression of KLF4	Zhang et al. (2019) and Zhou et al. (2020a)
	LincRNA-p21	Up in MPP^+^-induced cell	miR-1277-5p; miR-181; miR-625	Increase the expression of α-syn; Induce microglial activation; Inhibit MPP^+^-induced neuronal injury	Xu et al. (2018), Ye et al. (2018); and Ding et al. (2019)
	UCA1	Up in PD mouse and MPP^+^-induced cell	α-syn	Up-regulate expressions of α-syn; Regulate activation of the PI3K/AKT signaling pathway	Lu et al. (2018) and Cai et al. (2019)
	MALAT1	Down in PD mouse	miR-129; miR-124; miR-124–3p	Up-regulate expressions of α-syn; Promote the apoptosis; Down-regulate DAPK1 expression	Liu et al. (2017), Liu et al. (2020) and Xia et al. (2019)
	Mirt2	Down in PD SH-SY5Y cells	miR-101	Anti-inflammatory reaction	Han et al. (2019)
	GAS5	Up in PD mice model	miR-223–3p	Regulate the NLRP3 expression	Xu et al. (2020b)
	BACE1-AS	Up in PD rat model	α-syn; miR-34b–5p	Reduce α-syn expression; Downregulate BACE1 expression	Li Y. et al. (2020)
	NORAD	Down in PD SH-SY5Y cells	α-syn	Rescue MPP^+^-induced cellular destruction and apoptosis	Song et al. (2019)
	HOTAIR	Up in the PD mice and SH-SY5Y cell lines	LRRK2; miR-326; miR-874–5p	Up-regulate LRRK2 expression; Repress NLRP3 mediated pyroptosis; Contribute MPP^+^-triggered neuronal injury	Wang et al. (2017), Zhang Q. et al. (2020); and Zhang Y. et al. (2020)
	DAPK1	Up in SH-SY5Y cells	miR-124–3p	Promote the apoptosis	Lu et al. (2020)
	H19	Up in PD model mice	miR-585–3p; miR-301b–3p	Inhibit neurons apoptosis in MPTP-induced PD mice; Inhibit HPRT1 expression	Jiang et al. (2020) and Zhang Y. et al. (2020)
	TUG1	Up in PD model mice	miR-152–3p	Regulate PTEN expression	Zhai et al. (2020)
HD	HAR1	Down in HD mice model			Johnson et al. (2010)
	NEAT1	Up in HD patients	REST	Relate to regulation of *p*^53^ gene	Smeenk et al. (2011) and
	MEG3	Down in HD patients			Johnson (2012)
	HTTAS	Up in HD patients	HTT	Regulate HTT expression	Chung et al. (2011) and
	Abhd11os	Down in HD mice model			Francelle et al. (2015)
ALS	NEAT1_2	Up in the early ALS	RBP	Regulate the expression of ALS-related RBP	Nishimoto et al. (2013)

Nuclear paraspeckle assembly transcript 1 (NEAT1) is a widely expressed lncRNA in a variety of mammalian cell types (Wang et al., 2020b). Ke et al. have shown that NEAT1 expression was elevated in Aβ-induced SH-SY5Y neuroblastoma cells, whereas NEAT1 knockdown attenuated the Aβ-induced inhibition of cell viability, apoptosis promotion, and p-Tau levels. MiR-107 abundance is diminished in Aβ-treated SH-SY5Y neuroblastoma cells, and NEAT1 overexpression reverses Aβ-induced injury. Furthermore, NEAT1 acts as a decoy for miR-107, and NEAT1 aggravates Aβ-induced neuronal injury by sponging miR-107, thus suggesting a novel strategy for AD treatment (Ke et al., 2019). Spreafico et al. have observed that NEAT1 expression levels were elevated in the temporal cortex and hippocampus in patients with AD, and have further observed a strong positive correlation between NEAT1 expression and cyclin-dependent kinase 5 regulatory subunit 1 (CDK5R1), which was closely associated with AD onset and progression. Moreover, NEAT1 has a neuroprotective role in AD by compensating for increased CDK5R1 levels by sponging the miR-15/107 family (Spreafico et al., 2018). Zhao et al. (2019) have confirmed that NEAT1 was considerably up-regulated in an AD mouse model and identified a reversible regulatory relationship with miR-124. NEAT1 knockdown, or miR-124 over-expression has been found to elicit protective effects in a cellular AD model induced by Aβ. Moreover, NEAT1 has been suggested to function as a regulatory factor promoting AD development by modulating the miR-124/BACE1 axis; this pathway may be a potential novel therapeutic target for AD treatment (Zhao et al., 2019). In addition, NEAT1 interacts with NEDD4L and consequently promotes PTEN-induced putative kinase 1 (PINK1) ubiquitination and degradation, thus resulting in PINK1-dependent autophagy (Huang et al., 2020).

Expression of the lncRNA MEG3 has been found to be down-regulated in an Aβ_25–35_ induced AD rat model by Yi et al., who have suggested that MEG3 up-regulation improved memory and spatial learning abilities, decreased Aβ deposition in the hippocampus, and mitigated oxidative stress and inflammatory injury by inactivating the phosphatidylinositol 3-kinase and protein kinase B (PI3K/AKT) pathway in this model (Yi et al., 2019).

The lncRNA BC1 has been shown to be overexpressed in the brains of AD model mice. Exogenous BC1 expression in excitatory pyramidal neurons induces Aβ peptide accumulation, spatial learning, and memory impairments in these mice. Moreover, BC1 induces APP mRNA translation via its association with the Fragile X syndrome protein (FMRP). Thus, inhibiting the association of BC1 or BC1-FMRP in an AD mouse model decreases Aβ accumulation in the brain, and prevents spatial learning and memory loss (Zhang T. et al., 2018).

Massone et al. (2011) have described the novel lncRNA 17A, which was stably expressed in SH-SY5Y neuroblastoma cells, and was also expressed in the human brain and up-regulated in cerebral tissue from patients with AD. Moreover, 17A expression in neuroblastoma cells enhances Aβ secretion and Aβ _x-42_/Aβ _x-40_ peptide ratios by inducing responses to inflammatory stimuli.

Neuroinflammation plays a major role in AD (Calsolaro and Edison, 2016). MALAT1 expression has been found to be elevated in a PC12 cell line AD model. MALAT1 overexpression inhibits neuronal apoptosis, decreases IL-6 and TNF-α levels, promotes neurite outgrowth, and increases IL-10 level by sponging miR-125b; therefore, MALAT1 may have a neuroprotective role against AD (Ma et al., 2019). Li L. et al. (2020) have demonstrated that MALAT1 served as a sponge for mR-30b, thereby resulting in up-regulation of cannabinoid receptor 1 (CNR1) expression. Moreover, phosphorylation of PI3K and AKT was stimulated when MALAT1 or CNR1 is over-expressed. In addition, MALAT1 has been found to promote neuronal recovery after AD onset in a rat model, via the miR-30b/CNR1 network and PI3K/AKT signaling activation.

The lncRNA at the INK4 locus (lnc-ANRIL) has been identified in several neurological diseases with pathologies associated with inflammation and neural dysfunction (Zhou B. et al., 2020). In Aβ_1–42_ assaulted nerve growth factor (NGF)-stimulated PC12 cells, lnc-ANRIL is up-regulated, whereas lnc-ANRIL knockdown inhibits inflammatory cytokine expression and increases neurite outgrowth by regulating miR-125a expression (Zhou B. et al., 2020).

Oxidative stress has also been implicated in AD pathogenesis and progression (Tobore, 2019). Yue et al. have demonstrated that the expression of the lncRNA XIST was significantly up-regulated in *in vivo* and *in vitro* AD models. The silencing of lncRNA XIST negatively regulates miR-124 and positively regulates BACE1 expression in N2a cells, thus suggesting that lncRNA XIST may be a new potential target for AD treatment (Yue et al., 2020). Additionally, Wang X. et al. (2018) have shown that lncRNA XIST knockdown alleviated Aβ_25–35_-induced oxidative stress, toxicity, and apoptosis in primary cultured rat hippocampal neurons *via* miR-132 up-regulation. These observations underpin the potential of manipulating lncRNA XIST in AD treatment.

Cai et al. (2017) have revealed that the lncRNA RPPH1 was up-regulated in an AD mouse model, thereby up-regulating CDC42 expression and promoting hippocampal neuron dendritic spine formation. Gu et al. (2020b) have demonstrated that both lncRNA RPPH1 and miR-122 were up-regulated in an AD mouse model, and that lncRNA RPPH1 activated Wnt/β-catenin signaling, thus ameliorating Aβ induced neuronal apoptosis in SK-N-SH cells *via* direct targeting of miR-122. In addition, the overexpression of lncRNA RPPH1 down-regulates the endoplasmic reticulum stress (ERS) related proteins CHOP and GRP78, as well as cleaved caspase 12. Moreover, lncRNA RPPH1 directly targets miR-326 and counteracts its inhibitory effects on PKM2 expression, thereby attenuating the ERS status and apoptosis induced by Aβ_25–35_ (Gu et al., 2020a).

Other lncRNAs have been implicated in AD pathogenesis *via* several molecular pathways. The lncRNA-ATB was the first lncRNA found to be activated by transforming growth factor-β (TGF- β) (Saito et al., 2015). lncRNA-ATB promotes epithelial-mesenchymal transformation in gastric cancer by regulating TGF-β and the miR-200 family (Saito et al., 2015). In Aβ_25–35_-induced PC12 cells, and the cerebrospinal fluid (CSF) and serum from patients with AD, lncRNA-ATB expression levels are significantly elevated (Wang J. et al., 2018). Furthermore, Wang J. et al. (2018) have indicated that the suppression of lncRNA-ATB protected PC12 cells against Aβ_25–35_-induced neurotoxicity via regulation of the miR-200/ZNF217 axis, thereby providing new insights for AD prevention.

The lncRNA EBF3-AS is abundantly expressed in the brain and has been reported to be dysregulated in an AD mouse model (Gu et al., 2018). Gu et al. have shown that lncRNA EBF3-AS expression was up-regulated in the hippocampus in APP/PS1 mice. Knockdown of lncRNA EBF3-AS by siRNA inhibits Aβ_25–35_ induced apoptosis, thus suggesting that lncRNA EBF3-AS promotes AD neuronal apoptosis and indicating its potential as a new therapeutic target for AD treatment (Gu et al., 2018).

Inhibition of BC200, a highly expressed lncRNA in AD, may be an effective method for AD therapy (Li et al., 2018). Li et al. (2018) have established an AD cell model with overexpression of Aβ_1–42_ to investigate the effects of BC200 on cell viability and apoptosis. They have observed that BC200 knockdown suppressed BACE1 expression, thus suggesting that this may be a putative target for AD therapeutics.

The lncRNA SNHG1 has been reported to show increased expression in SH-SY5Y and human primary neuronal (HPN) cells, and lncRNA SNHG1 knockdown partially reverses the effects of Aβ_25–35_ treatment on apoptosis, cell viability, and caspase-3 activity (Wang et al., 2019). In addition, SNHG1 appears to function as a competing endogenous RNA (ceRNA) for miR-137, which negatively regulates kringle containing transmembrane protein 1 (KREMEN1) expression by targeting its 3′ untranslated region. Wang et al. (2019) have indicated that SNHG1 knockdown exerted neuronal protective effects by repressing KRENEN1 and by acting as a ceRNA for miR-137, in an *in vitro* AD cell model. Gao et al. (2020) have also suggested that SNHG1 expression was up-regulated in an *in vitro* AD cell model. Thus, SNHG1 may promote cell injury by regulating the miR-361-3p/ZNF217 axis; this knowledge may provide a theoretical basis for AD molecular therapy.

The lncRNA taurine up-regulated gene 1 (TUG1) was first recognized in 2005 as an important element in retinal development in rodents. This lncRNA has also been shown to participate in oncogenic processes by sponging miRNAs (Li et al., 2016). Li X. et al. (2020) have observed that TUG1 expression was up-regulated in an AD mouse model, whereas TUG1 knockdown improved memory and spatial learning ability, decreased pathological neuronal injury, and inhibited neuronal apoptosis by elevating miR-15a levels and suppressing Rho-associated kinase 1 (ROCK1) expression in an AD mouse model.

Linc00507 is significantly elevated in the brain in APP/PS transgenic mice and in SH-SY5Y cells (Yan et al., 2020). It directly binds miR-181c-5p and regulates the expression of microtubule-associated protein Tau (MAPT) and tau-tubulin kinase-1 (TTBK1) as a ceRNA. In addition, linc00507 mediates tau protein hyperphosphorylation by activating the P25/P35/GSK3β signaling pathway sponging miR-181c-5p (Yan et al., 2020).

IAβ_1–42_-treated SH-SY5Y cells show increased expression of the lncRNA SOX21 antisense RNA1 (SOX21-AS1) and decreased expression of miR-107 (Xu et al., 2020a). SOX21-AS1 acts as a sponge for miR-107, whereas silenced SOX21-AS1 attenuates Aβ_1–42_, mediated p-Tau levels, and SH-SY5Y cell viability by sponging miR-107, thus suggesting a possible role in AD therapeutics (Xu et al., 2020a).

Expression of the lncRNA brain-derived neurotrophic factor (BDNF)-AS is increased by Aβ_25–35_-induced neurotoxicity in PC12 cells, thus inhibiting BDNF expression and inducing apoptosis in PC12 cells (Guo et al., 2018). Furthermore, silenced lncRNA BDNF-AS up-regulates BDNF expression and cell viability, inhibits apoptosis, and exerts protective functions in Aβ_25–35_-induced neurotoxicity in PC12 cells (Guo et al., 2018).

Zhu et al. (2019) have shown that linc00094 increased in Aβ_1–42_ co-incubated with microvascular endothelial cells in an *in vitro* blood-brain barrier model. Silencing of linc00094 inhibits endophilin-1 expression by up-regulating miR-224-4p/miR-497-5p, increasing ZO-1, occludin, and claudin-5 expression, and ultimately alleviating blood-brain barrier permeability in an AD microenvironment. These data suggest that the linc00094/miR-224–5p (miR-497-5p)/endophilin-1 axis may have a crucial role in regulating blood-brain barrier permeability in the AD microenvironment (Zhu et al., 2019).

### PD-Associated lncRNAs

PD, a common NDD caused by the gradual loss of dopaminergic neurons in the substantia nigra striatum of the midbrain, is characterized by static tremors, dyskinesia, and an abnormal postural gait. In severe cases, dementia appears, but the exact cause remains unknown.

Autophagy has been shown to be associated with the central mechanisms underlying PD. In a recent study, lncRNA SNHG1 has been reported to be significantly elevated in PD (Qian et al., 2019). The authors have demonstrated that lncRNA SNHG1 knockdown promoted autophagy and prevents 1-methyl-4-phenylpyridinium (MPP+)-induced cell death, whereas SNHG1 down-regulation further attenuated MPP+-induced decreases in LC3-II levels and cytotoxicity *via* the miR-221/222/p27/mTOR pathway. Over-expression of the lncRNA SNHG1 also aggravates MPP+-induced cytotoxicity in SH-SY5Y cells by regulating PTEN/AKT/mTOR signaling *via* miR-153–3p sponging. These data suggest that SNHG1 may serve as a therapeutic target for PD neuroprotection and treatment (Qian et al., 2019; Zhao et al., 2020a).

NEAT1 is closely associated with pathological changes in the CNS. Yan et al. have observed that NEAT1 expression was elevated in a PD mouse model (Yan et al., 2018). Moreover, NEAT1 positively regulates PINK1 protein levels by inhibiting degradation of the PINK1 protein, whereas NEAT1 knockdown effectively suppresses *in vivo* 1-methyl-4-phenyl-1,2,3, 6-tetrahydropyridine (MPTP)-induced autophagy, thus alleviating dopaminergic neuronal injury. These data indicate that NEAT1 promotes MPTP-induced autophagy in PD via PINK1 stabilization (Yan et al., 2018). NEAT1 down-regulation also decreases α-syn-induced activation of NLRP3 inflammation by inhibiting GJB1 expression via miR-1301-3p sponging (Sun et al., 2020). Additionally, up-regulated NEAT1 appears to contribute to MPP^+^-induced neuronal injury via the NEAT1-miR-1277-5p-ARHGAP26 ceRNA pathway (Zhou et al., 2020a). NEAT1 knockdown also suppresses MPP^+^-induced inflammation, apoptosis, and cytotoxicity in SK-N-SH cells by regulating both miR-212-5p and RAB3IP expression. These data suggest a possible therapeutic strategy based on NEAT1 in patients with PD (Liu et al., 2020).

HAGLROS expression has been found to be elevated in an MPTP-induced PD mouse model and in MPP+-induced SH-SY5Y cells (Peng et al., 2019). HAGLROS regulates autophagy and apoptosis in MPP+ induced SH-SY5Y cells by sponging miR-100, whereas up-regulated HAGLROS appears to contribute to PD development by inhibiting apoptosis and autophagy, effects that might be achieved through regulation of the miR100/ATG10 axis and PI3K/AKT/mTOR pathway (Peng et al., 2019).

Fan et al. (2020) have shown that the lncRNA BDNF-AS was also dysregulated in PD. lncRNA BDNF-AS was up-regulated in both *in vivo* and *vitro* PD models. In addition, lncRNA BDNF-AS knockdown has been found to elevate SH-SY5Y cell viability, and inhibit autophagy and apoptosis in an MPTP-induced PD model by regulating miR-125b-5p, thus suggesting that lncRNA BDNF-AS may serve as a potential therapeutic target for PD.

Pathological changes in PD are mediated by the formation of Lewy bodies (LB). Because α-syn is the main component of LB, α-syn alterations may affect PD pathogenesis. SNHG14 expression is elevated in the brain tissue in PD mice (Zhang et al., 2019). Moreover, miR-133b down-regulates α-syn expression by targeting its mRNA 3′ untranslated region, whereas SNHG14 has a reversible regulatory relationship with miR-133b. The authors have also demonstrated that SNHG14 silencing mitigated dopaminergic neuron injury by down-regulating α-syn through targeting miR-133b, thus potentially ameliorating PD symptoms (Zhang et al., 2019). Additionally, SNHG14 knockdown appeared to protect SK-N-SH cells against MPP+-induced cytotoxicity through up-regulation of miR-214-3p. KLF4 is a direct target of miR-214-3p, and SNHG14 appears to regulate KLF4 expression by acting as a miR-214-3p sponge and therefore may be a promising target for PD intervention (Zhou et al., 2020b).

The lincRNA-p21 has been reported to be elevated in PD. Up-regulated lincRNA-p21 sponges miR-1277-5p and indirectly increases α-syn expression, thus suppressing SH-SY5Y viability and activating apoptosis in these cells. These data suggest that lincRNA-p21 may serve as a novel target for PD (Xu et al., 2018). In addition, lincRNA-p21 competitively binds the miR-181 family and induces microglial activation via the miR-181/PKC-δ pathway (Ye et al., 2018). Moreover, lincRNA-p21 inhibits MPP+-induced neuronal injury by sponging miR-625 and up-regulating TRPM2 in SH-SY5Y cells. These data have increased the understanding of PD pathogenesis (Ding et al., 2019).

UCA1 may also regulate PD progression by modulating α-syn expression (Lu et al., 2018). UCA1 and α-syn are highly expressed in brain tissue in a PD mouse model and in MPP+-induced SH-SY5Y cells. UCA1 overexpression considerably up-regulates the mRNA and protein expression of α-syn, whereas UCA1 knockdown decreases caspase-3 activity and MPP+-induced neuronal apoptosis in SH-SY5Y cells (Lu et al., 2018). Similarly, Cai et al. have shown that UCA1 down-regulation increased dopamine levels, inhibited apoptosis and oxidative stress in substantia nigra neurons, and decreased neuroinflammatory responses in a rat model of PDs. Furthermore, UCA1 down-regulation inhibits the activation of the PI3K/AKT signaling pathway in the substantia nigra in a rat model of PD, thus suggesting a protective mechanism toward dopaminergic neurons in a rat PD model (Cai et al., 2019).

MALAT1 has also been implicated in PD pathogenesis. The expression of MALAT1 has been found to be diminished in a PD mouse model, whereas its overexpression up-regulates α-syn levels by inhibiting miR-129 expression (Xia et al., 2019). MALAT1 also promotes apoptosis by sponging miR-124 in PD mouse models, thus providing a potential therapeutic foundation for the clinical use of MALAT1 against PD (Liu et al., 2017).

Neuroinflammation plays an important role in PD pathogenesis. SNHG1 has been found to be elevated in brain specimens from patients with PD and in MPP+-induced SH-SY5Y cells. Cao et al. have observed that SNHG1 functioned as a ceRNA for miR-7, thus regulating nod-like receptor protein 3 (NLRP3) expression and activating the NLRP3 inflammasome pathway. These findings suggest that SNHG1 promotes neuroinflammation in PD pathogenesis by modulating the miR-7/NLRP3 pathway (Cao et al., 2018).

The lncRNA Mirt2 has also been shown to decrease inflammatory reactions in numerous cell types (Han et al., 2019). Mirt2 has been suggested to elevate SH-SY5Y cell resistance to inflammation, whereas Mirt2 overexpression generates anti-inflammatory properties by suppressing miR-101 expression and inhibiting the TNF-α-triggered NF-κB/p38MAPK pathway (Han et al., 2019).

The lncRNA GAS5 has also been shown to be associated with inflammatory responses. GAS5 has been found to be up-regulated in a PD mouse model and to positively regulate NLRP3 expression by competitively sponging miR-223-3p. These findings suggest that GAS5 accelerates PD progression by targeting the miR-223-3p/NLRP3 axis (Xu et al., 2020b).

Oxidative stress injury to dopaminergic neurons is another important pathological mechanism in PD pathogenesis and progression. The lncRNA BACE1-AS has been implicated in PD pathogenesis through regulating dopaminergic neuron oxidative stress injury. Li Y. et al. (2020) have observed that BACE1-AS levels are elevated in the substantia nigra of a rat model of PD, whereas down-regulated BACE1-AS decreases levels of α-syn, inducible nitric oxide synthase, and glutamine levels. In addition, down-regulated BACE1-AS has been found to repress apoptosis and oxidative stress injury in the substantia nigra neurons in a rat model of PDs by up-regulating miR-34b-5p.

In MPP+-induced PD-like cytotoxicity, NORAD expression levels are down-regulated in SH-SY5Y cells. In contrast, up-regulated NORAD protects against MPP+-induced cytotoxicity in these cells by rescuing MPP+-induced cellular destruction and apoptosis, as well as decreasing caspases 3/7, reactive oxygen species, and lactate dehydrogenase levels (Song et al., 2019).

Hox transcript antisense intergenic RNA (HOTAIR) is an approximately 2.2 kb lncRNA transcribed from the HOXC locus (Wang et al., 2017). The transcript is up-regulated in a PD mouse model and in SH-SY5Y cells pretreated with MPP+ and is believed to be involved in PD pathogenesis through up-regulating LRRK2 expression (Wang et al., 2017). HOTAIR is highly expressed in a variety of tumors, in which it may promote malignant biological behavior, but its role in PD pathogenesis remains unclear. Zhang Q. et al. (2020) have demonstrated that HOTAIR was up-regulated in PD model mice and MPP+ induced SH-SY5Y cells. HOTAIR knockdown notably ameliorates PD symptoms *in vivo*. In addition, HOTAIR silencing significantly inhibits neuronal damage by repressing NLRP3 mediated pyroptosis activation via regulation of the miR-326/ELAVL1 axis in PD mice (Zhang Q. et al., 2020). Moreover, HOTAIR appears to aggregate MPP+-triggered neuronal injury by sponging miR-874-5p; therefore, modulating HOTAIR expression may provide a therapeutic mechanism for PD treatment (Zhao et al., 2020b).

DAPK1, first identified during γ-interferon-induced programmed cell death, is a key factor in the CNS, including PD etiology (Lu et al., 2020). DAPK1 is up-regulated and negatively correlated with miR-124-3p levels in SH-SY5Y cells treated with MPP+. MALAT1 regulates miR-124-3p, whereas MALAT1 knockdown ameliorates animal behavioral changes and decreases apoptosis by up-regulating miR-124-3p and down-regulating DAPK1 in an MPTP induced PD mouse model. These data implicate the MALAT1/miR-124-3p/DAPK1 signaling cascade in PD pathogenesis (Lu et al., 2020). Additionally, MALAT1 is highly expressed in the brains of MPTP-induced PD model mice (Cai et al., 2020). Knockdown of MALAT1 inhibits elevated NRF2 expression, thereby inhibiting inflammasome activation and the production of reactive oxygen species in PD mice (Cai et al., 2020).

The lncRNA H19 is associated with PD progression (Zhang Y. et al., 2020); lncRNA H19 inhibits neuronal apoptosis in MPTP-induced PD mice and MPP+ treated neuroblastoma cells by regulating the miR-585-3p/PIK3R3 pathway (Zhang Y. et al., 2020). In addition, H19 over-expression protects against dopaminergic neuronal loss in this PD pathway by impairing the miR-301b-3p-targeted inhibition of HPRT1 expression. These data suggest a potential theoretical strategy for PD treatment by targeting lncRNAs (Jiang et al., 2020).

TUG1 expression is up-regulated and miR-152-3p is down-regulated in a PD mouse model (Zhai et al., 2020). TUG1 sponges and regulates miR-152-3p expression, whereas miR-152-3p negatively regulates PTEN expression. Therefore, TUG1 knockdown exerts a protective effect against PD via the miR-152-3p/PTEN pathway (Zhai et al., 2020).

### HD-Related lncRNAs

HD is a rare autosomal dominant neurodegenerative disorder characterized by the loss of neurons in the striatum and cerebral cortex. The Huntingtin (HTT) protein has a CAG repeat sequence in the first exon, which encodes polyglutamine. Excessive amplification of the CAG repeat in gene coding regions causes polyglutamine chain amplification in the protein and results in disease. Wild-type *Huntingtin* regulates the nuclear translocation of RE1-silencing transcription factor (REST), whereas mutated *Huntingtin* causes abnormal REST nuclear translocation, thereby leading to abnormal expression of REST downstream target genes, including protein-coding genes and ncRNAs (Shimojo, 2008). After investigating the brain expression profiles in patients with HD, Johnson et al. (2010) have observed that expression of the lncRNA Human Accelerated Region 1 (HAR1) significantly decreased in the striatum, because REST localized to HAR1 loci via specific DNA regulatory elements, thereby inhibiting HAR1 transcription. Smeenk et al. (2011) have found that the lncRNA NEAT1 was up-regulated in patients with HD, and plays a different role with REST in the pathogenesis of HD. MEG3 has also been found to contain binding sites for REST within 10 kb of its transcription start site, thus suggesting that MEG3 may also be a REST target. Therefore, the substantial down-regulation of MEG3 in the brain tissues of patients with HD may be partly due to REST suppression (Johnson, 2012).

LINC0341 and RPS20P22 are both up-regulated in patients with HD, whereas LINC00342 is down-regulated, thereby revealing changes in neurological specific lncRNA expression patterns in patients with HD (Johnson, 2012). In the HD cell model, HTTAS overexpression has been found to decrease HTT transcription levels; however, siRNA knockdown of HTTAS v1 increases HTT transcription levels. These data provide strong evidence of the existence of an HTT antisense strand (Chung et al., 2011).

Studies by Francelle et al. (2015) have shown that Abhd11os expression in an HD mouse model was significantly diminished. In contrast, overexpression of Abhd11os elicited neuroprotective effects against the mutant HTT N-terminal fragment; however, Abhd11os knockout also elicited cytotoxic effects. In addition, the loss of lncRNA Abhd11os promotes vulnerability in the HD striatum (Francelle et al., 2015).

Sunwoo et al. (2017) have analyzed lncRNA expression patterns in HD *via* gene chips and found that lncRNA NEAT1 was highly expressed in the brains of patients with HD and in R6/2 mice. Furthermore, NEAT1 plays a neuroprotective role in HD; however, the specific mechanism remains unclarified (Sunwoo et al., 2017).

### ALS-Related lncRNAs

ALS is a fatal NDD and is the most common form of motor neuron disease. Although the pathogenesis of ALS is not fully elucidated, its characteristic pathological manifestations are progressive exacerbation of limb weakness, muscle atrophy, and pyramidal tract signs, accompanied by bulbar palsy, dysphagia, and respiratory muscle involvement (Al-Chalabi and Hardiman, 2013; Bonafede and Mariotti, 2017). Approximately 5%–10% of patients with ALS have a family history of the disease, but the genes associated with ALS remain to be fully characterized (Sunwoo et al., 2017).

Expression of the lncRNA NEAT1 is significantly up-regulated in early ALS motor neurons. NEAT1/paraspeckle-related defects are closely associated with NDD occurrence. Previous studies have identified FUS as a disease-related ALS gene; FUS directly binds NEAT1 and localizes to nuclear paraspeckles. In *in vivo* studies on ALS, FUS has been shown to have undergone mutation, thus resulting in its relocalization from the nucleus to the cytoplasm and in nuclear paraspeckle functional defects. Moreover, *in vitro* experiments have shown that siRNA-mediated FUS interference hinders nuclear paraspeckle formation. Data from Nishimoto et al. have shown that NEAT1_2 was minimally expressed in the motor neurons of healthy people but highly expressed in the motor neurons of people with early-stage ALS, thus suggesting its utility as a biomarker for early ALS diagnostics. In addition, NEAT1_2 may serve as the backbone of RNA and RNA-binding protein (RBP) in the nuclei in ALS motor neurons, thereby regulating the expression of ALS-related RBP and exemplifying the roles of lncRNAs in ALS pathological processes (Nishimoto et al., 2013).

### Circulating lncRNAs

lncRNAs participate in multiple biological processes and are associated with the occurrence and progression of several NDDs. In recent years, the detection of circulating lncRNAs in body fluids from patients with NDD has been demonstrated to be an effective diagnostic method (Table 2). Abnormal lncRNA expression is found not only in tissues and cells but also in various body fluids including the blood, urine, saliva, and CSF. Ren et al. have found that after repeated freezing and thawing, even in the presence of ribonucleases, lncRNAs from circulating plasma remained stable (Ren et al., 2013).

**Table 2:** Circulating lncRNA associated with NDDs.

Circulating	lncRNAs	Changes	Function	Reference
Plasma	BACE1–AS	High in plasm from 45 full-AD patients	AD progression and diagnosis	Fotuhi et al. (2019)
Plasma	BACE1–AS	High in plasm exosome from 72 AD patients	AD diagnosis	Wang D. et al. (2020)
Plasma	BACE1	High in plasm from 88 AD patients	AD diagnosis	Feng et al. (2018)
CSF; plasma	MALAT1	Low in CSF and plasm from 120 AD patients	AD progression and diagnosis	Zhuang et al. (2020)
Blood	MKRN2–42:1	Low in peripheral blood from 32 PD patients	PD diagnosis and disease monitoring	Wang et al. (2020)
Leukocytes	lnc-MOK–6:1; HOTAIRM1; RF01976.1–201; AC131056.3–001	High in leukocytes from 72 PD patients	PD diagnosis	Fan et al. (2019)
Serum	TUG1	High in serum from 97 PD patients	PD progression and diagnosis	Cheng et al. (2020)
Blood	NEAT1	High in blood from 43 PD patients	PD diagnosis	Boros et al. (2020)

Gene chips and next-generation sequencing technologies have established an extensive archive of transcripts from the human genome. On the basis of these technologies, research on lncRNAs has rapidly developed. Several circulating lncRNAs have been shown to be effective in clinical diagnostic and prognostic applications, and have served as robust biomarkers for NDDs. Single or combined circulating lncRNAs have also demonstrated diagnostic performance comparable or superior to that of conventional NDD markers. In the next section, we further explore the potential of lncRNAs as diagnostic biomarkers for NDD.

### Potential of lncRNAs as Diagnostic Ndd Biomarkers

BACE1-AS is up-regulated in the AD brain and potentially the bloodstream (Fotuhi et al., 2019). Fotuhi et al. have evaluated the AD diagnostic value of BACE1-AS in 45 patients with AD and 36 healthy controls. BACE1-AS levels were lower in patients with pre-AD, but higher in patients with full-AD, than in healthy controls. Receiver operating characteristic curve analysis has shown that BACE1-AS levels can be used to discriminate among pre-AD, full-AD, and healthy control samples with high sensitivity and specificity, thereby highlighting its potential utility as a biomarker for AD progression and diagnosis (Fotuhi et al., 2019).

Wang D. et al. (2020) have assessed whether plasma exosomal lncRNA levels in combination with image data from the entorhinal cortex and hippocampus could be used as a combinatorial AD biomarker, in a study on 72 patients with AD and 62 healthy controls. The plasma exosomal BACE1AS levels were significantly higher in the patients with AD than the healthy controls. Receiver operating characteristic curve analysis indicated an area under the curve of 0.761 for BACE1AS, with 87.5% sensitivity and 61.3% specificity. In addition, series parallel testing integrating BACE1AS levels with right entorhinal cortex volume and thickness have shown significantly increased specificity and sensitivity, at 96.15% and 90.91%, respectively.

In a study involving 88 patients with AD, Feng et al. have reported similar data: the plasma BACE1 levels were significantly elevated in patients with AD and distinguished patients with AD from healthy controls (88% specificity for AD). These observations suggest that BACE1 may be a potential candidate biomarker for predicting AD (Feng et al., 2018).

Zhuang et al. (2020) have detected and explored the clinical value of MALAT1 levels in CSF and plasma samples from 120 patients with AD. The MALAT1 levels were down-regulated in both the CSF and plasma, a finding associated with lower disease severity. The CSF MALAT1 levels also predicted MMSE score declines at years 1, 2, and 3 in patients with AD (Zhuang et al., 2020).

Similar studies have been conducted in patients with PD. Wang Q. et al. (2020) have performed next-generation sequencing of exosomal lncRNAs in peripheral blood samples from 32 patients with PD. Levels of lnc-MKRN2-42:1 were significantly lower in patients with PD. In addition, lnc-MKRN2-42:1 was positively associated with MDS-UPDRS III scores in patients with PD, thus suggesting that this lncRNA may be associated with PD occurrence and development. Lnc-MKRN2-42:1 has therefore been proposed as a biomarker for PD diagnosis and disease monitoring.

In a cohort of 72 patients with PD and 22 healthy controls, Fan et al. (2019) have identified 122 differentially expressed lncRNAs in the circulating leukocytes of patients with PD. Four lncRNAs (lnc-MOK-6:1, HOTAIRM1, RF01976.1-201, and AC131056.3-001) were further confirmed to be up-regulated in these patients. Moreover, the dysregulated lncRNAs AC131056.3-001 and HOTAIRM1 may contribute to PD pathogenesis by enhancing apoptosis in dopaminergic neurons.

Cheng et al. (2020) have evaluated the diagnostic value of serum TUG1 levels for PD in a case-control study including 97 patients with PD and 84 healthy controls. The TUG1 levels were significantly higher in patients with PD, thus suggesting that TUG1 could adequately distinguish patients with PD from healthy controls, with an area under the curve of 0.902. In addition, serum TUG1 was positively correlated with UPDRS levels, thus suggesting that TUG1 may be a possible target for PD early diagnosis and therapeutic intervention. Moreover, NEAT1 levels have been found to be up-regulated in PD blood samples, thus suggesting its potential as a PD biomarker (Boros et al., 2020).

The lncRNAs entering the circulatory system are stable in the blood or other body fluids (urine, saliva); thus, lncRNAs could be easily detected in these samples. However, the current detection of circulating lncRNAs still has many deficiencies and cannot be directly used in the early diagnosis of various NDDs in clinical practice. First, a consensus is lacking regarding the reference genes for circulating lncRNAs, thus preventing the determination of which genes are stable and are suitable reference genes. Further research remains to be performed on how to use appropriate reference genes to calculate the expression of circulating lncRNA and how to improve the accuracy of detection. Second, many circulating lncRNAs are difficult to detect in the circulatory system despite their lower expression than that of other circulating nucleic acids after pre-amplification. Therefore, the detection of circulating lncRNA must be improved in the selection and establishment of methods. Third, circulating lncRNAs with differential expression have been found to lack specificity for individual NDDs. For example, NEAT1 shows differential expression in AD, PD, and ALS. Therefore, combined detection based on multiple lncRNAs and combined diagnostic application with traditional serum biomarkers should greatly improve the diagnostic value and will be an important direction for development in the future. Current research on lncRNAs is primarily at the expression level. Similarly, clinical sample numbers are inadequate, and an incomplete understanding of the regulatory mechanisms precludes direct application in clinical practice. The lncRNA detection technologies and methods, including sample preparation, extraction methods, and internal reference selection, must be expanded and improved to meet these requirements.

### Clinical Applications and Limitations of lncRNAs

Unlike mRNA molecules, lncRNAs are functional molecules. Their highly specific expression suggests that lncRNAs may serve as good biomarkers. In addition, lncRNA use in diagnostics and research on the therapeutic targeting of lncRNAs has received substantial attention. For pathogenic lncRNAs, the following blocking/silencing methodologies are primarily used. (1) Silencing lncRNA: (i) RNA interference to inhibit lncRNA expression; (ii) antisense oligonucleotides to block lncRNA activity; and (iii) RNaseH to induce lncRNA degradation. (2) Small molecule inhibitors that mask lncRNA binding sites and interacting proteins to block functions. (3) Small molecule inhibitors that bind lncRNAs, thus affecting folding and conformation.

For lncRNAs with neuroprotective or therapeutic effects, modern biological techniques can facilitate their increased expression at specific locations in the CNS for therapeutic purposes. For example, Espinoza et al. (2020) have used an adenovirus 9-mediated delivery system to increase SINEUP expression in the striatum in wild-type mice, thus indicating endogenous glial cell line-derived neurotrophic factor (GDNF) protein expression and potentiation of the DA system’s functions. In addition, SINEUP-GDNF has been found to ameliorate motor deficits and neurodegeneration of DA neurons in a PD neurochemical mouse model.

However, the current understanding of lncRNAs limits their clinical application. In addition, although lncRNAs are considered tissue and disease-specific, this specificity is not strong. For example, TUG1 is dysregulated in not only NDDs but also cancer and other diseases. Moreover, the viral system used to deliver lncRNA may have low efficiency in targeting NDDs, thus limiting the possibilities for using lncRNA as therapeutic strategy in the treatment of NDDs. Further research is needed to pursue these directions in the future.

### Conclusions

The recent discovery of lncRNA has revolutionized the understanding of this anthropogenic group. lncRNA research remains in its infancy, and nomenclature schemes, structures, properties, and functional aspects remain to be standardized. In recent years, researchers have observed that lncRNAs played important roles in CNS development and NDD via epigenetic, translational, and posttranslational modifications. With the continued in-depth study of lncRNAs, specific lncRNAs are expected not only to be helpful in elucidating disease pathophysiological processes but also to become novel biomarkers and drug targets. Although very little is known about lncRNA function, and much bench research awaits, continued lncRNA exploration will doubtlessly provide new directions for NDD treatment and clinical drug development.

### Author Contributions

MZ and PH drafted and revised the manuscript. ZB drafted and modified the figures and tables. All authors approved the final version of the manuscript and have agreed to be accountable for all aspects of the work, to ensure that questions associated with the accuracy or integrity of any part of the work are appropriately investigated and resolved. All authors contributed to the article and approved the submitted version.

## Conflict of Interest

The authors declare that the research was conducted in the absence of any commercial or financial relationships that could be construed as a potential conflict of interest.

## Publisher’s Note

All claims expressed in this article are solely those of the authors and do not necessarily represent those of their affiliated organizations, or those of the publisher, the editors and the reviewers. Any product that may be evaluated in this article, or claim that may be made by its manufacturer, is not guaranteed or endorsed by the publisher.
